# Electrodeposition of Stable Noble-Metal-Free Co-P Electrocatalysts for Hydrogen Evolution Reaction

**DOI:** 10.3390/ma16020593

**Published:** 2023-01-07

**Authors:** Jeongwon Kim, Yu Jin Jang, Yoon Hee Jang

**Affiliations:** 1 Advanced Photovoltaics Research Center, Korea Institute of Science and Technology (KIST), Seoul 02792, Republic of Korea; 2 Convergence Research Center for Energy and Environmental Sciences, Sungkyunkwan University (SKKU), Suwon 16419, Republic of Korea

**Keywords:** cobalt phosphide, electrodeposition, hydrogen evolution reaction, electrocatalyst, long-term stability

## Abstract

Hydrogen production via water splitting has been extensively explored over the past few decades, and considerable effort has been directed toward finding more reactive and cost-effective electrocatalysts by engineering their compositions, shapes, and crystal structures. In this study, we developed hierarchical cobalt phosphide (Co-P) nanosphere assemblies as non-noble metal electrocatalysts via one-step electrodeposition. The morphologies of the Co-P nanostructures and their electrocatalytic activities towards the hydrogen evolution reactions (HER) were controlled by the applied potentials during electrodeposition. The physicochemical properties of the as-prepared Co-P nanostructures in this study were characterized by field-emission scanning electron microscopy, X-ray photoemission spectroscopy and X-ray diffraction. Linear sweep voltammetry revealed that the Co-P grown at −0.9 V showed the best HER performance exhibiting the highest electrochemical active surface area and lowest interfacial charge transfer resistance. The Co-P electrocatalysts showed superior long-term stability to electrodeposited Pt, indicating their potential benefits.

## 1. Introduction

Water splitting is the first step in converting solar energy into chemical energy in nature [1,2]. Researchers have emulated this reaction in the past few decades to efficiently produce hydrogen fuels [3,4]. Water splitting (2H_2_O → O_2_ + 2H_2_) is a non-spontaneous reaction (ΔG = 237.1 kJ/mol) [5,6] that occurs through two half-reactions: an oxygen evolution reaction (OER; 2H_2_O → O_2_ + 4H^+^ + 4e^−^, 1.23 V vs. standard hydrogen electrode (SHE)) and hydrogen evolution reaction (HER; 4H^+^ + 4e^−^ → 2H_2_, 0 V vs. SHE) in acidic media [7]. The specific thermodynamic potential of water splitting (1.23 V) restricts the availability of semiconductors [5,8], and only a few resources are substantially active toward water electrolysis. Ru, Ir, and Pt-based materials have demonstrated the best performance for the OER and HER in terms of current density and overpotential levels [9,10]. However, transition metal (Fe, Co, Ni, Mo, and W) carbides, nitrides, and chalcogenides have also been widely investigated as alternatives to expensive and scarce noble-metal-based electrocatalysts [11,12,13].

Cobalt phosphide (Co-P), which is representative of a transition metal phosphide (TMP) family, has attracted considerable attention as an efficient non-noble metal electrocatalyst [14]. The difference in electronegativities of Co and P is derived from the partial negative charges on P atoms; thus, protons from water are initially adsorbed onto the electrocatalytic surfaces [15]. Density functional theory calculations from previous studies showed that the Gibbs free energy of hydrogen adsorption (ΔG_H*_) on Co-P was negative, indicating that the evolution of clean and renewable H_2_ fuels on Co-P could be definitive [16,17]. Heteroatom doping, that is, the incorporation of non-metals (O and B) [18,19] or metals (Mo, Ni, and Fe) [20,21,22,23,24], into Co-P was also effective in improving the HER activities because it altered the electronic structure of the pure Co-P compound and optimized ΔG_H*_ [14]. Because the geometry and dimensions of Co-P affect its electrocatalytic activities, the synthesis techniques that govern the structural properties of Co-P are critical. Therefore, fabricating Co-P electrocatalysts using various precursors, such as solid Co, organometallic Co complexes, and Co salts, has been attempted in diverse solvents and temperature conditions, followed by phosphidation [14]. As mentioned above, recent research indicates that a new approach to manipulating the structural and compositional properties of Co-P should be developed for highly efficient TMPs for the HER.

Here, we employed a one-step electrodeposition technique, a facile and low-cost method to directly grow multicomponent electrocatalysts on a wide range of solid substrates [25,26,27] for the preparation of hierarchical Co-P nanospheres on indium tin oxide (ITO)-coated glasses. The results obtained from field-emission scanning electron microscopy (FE-SEM) measurements confirmed that the applied voltages for the electrodeposition procedure were the key to controlling the morphologies and the coverages of the self-supported Co-P nanostructures. The linear sweep voltammogram showed that the Co-P nanostructure grown at a potential of −0.9 V exhibited the lowest overpotential for HER at a current density of 10 mA cm^−2^ in acidic media compared with that of other Co-P electrocatalysts prepared at higher applied potentials (that is, −1.0, −1.1, and −1.2 V), which was predominantly attributed to the enhanced charge transfer behavior and a higher electrochemical active surface area (ECSA). The electrodeposited Co-P showed better stability towards the HER over time than the noble metal Pt electrocatalysts.

## 2. Materials and Methods

### 2.1. Electrochemical Deposition of Electrocatalysts

**Figure 1:**
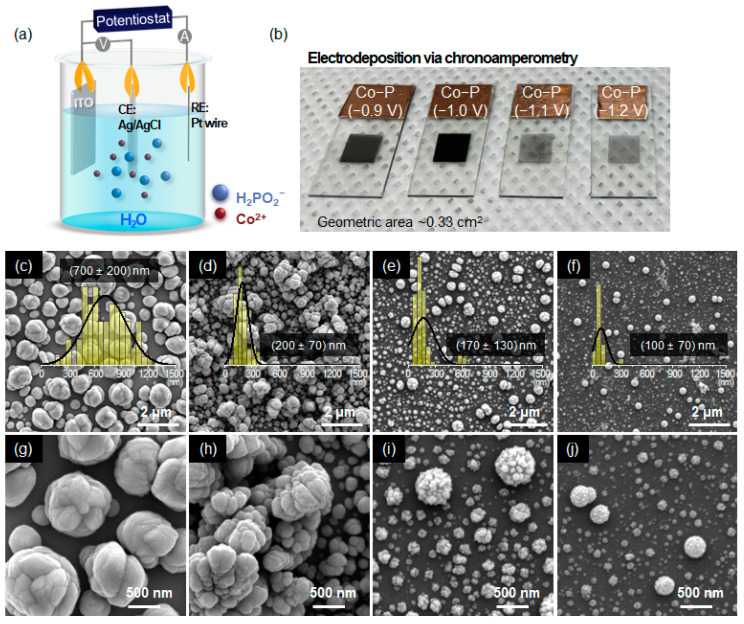
(**a**) Schematic of the Co-P nanocluster generation by electrodeposition. (**b**) Photographs and (**c**–**j**) SEM images of the resulting Co-P nanostructures on ITO which were grown at various applied potentials: (**c**,**g**) −0.9 V, (**d**,**h**) −1.0 V, (**e**,**i**) −1.1 V, and (**f**,**j**) −1.2 V (inset: size distribution histogram of nanoclusters).

As shown in Figure 1a, a potentiostat (AMETEK Princeton Applied Research, PARSTAT MC) with a three-electrode configuration was employed for the electrodeposition of Co-P and Pt electrocatalysts. Ag/AgCl in 3 M KCl(*l*) and Pt wire were used as the reference, and counter electrodes, respectively, and ITO-coated glass was utilized as a working electrode in an aqueous solution of 0.025 M CoSO_4_·7H_2_O, 0.5 M NaH_2_PO_2_·H_2_O, and 0.025 mM CH_3_COONa for Co-P or 0.5 mM of H_2_PtCl_6_·6H_2_O for Pt electrocatalysts. Potentials of −0.9 to −1.2 V (with respect to the Ag/AgCl electrode) for Co-P or −0.35 V (vs. Ag/AgCl) for Pt electrocatalysts was applied for 10 min.

### 2.2. Material Characterizations

The morphologies of the Co-P and Pt electrocatalysts were investigated by FE-SEM at an acceleration voltage of 10 kV (FEI, Inspect F, Hillsboro, OR, USA). The chemical composition of the Co-P hybrid nanostructures was examined using X-ray photoemission spectroscopy (XPS) (Nexas system, Thermo Fisher Scientific, Waltham, MA, USA) equipped with a monochromatic Al Kα photon source (1486.6 eV, 72 W, 12 kV). X-ray diffraction (XRD) patterns were collected by an X-ray diffractometer (Dmax2500/PC, Rigaku, Tokyo, Japan) using Cu Kα radiation (λ = 1.5406 Å) at a scanning rate of 2°/min.

### 2.3. Electrochemical Measurement

The HER activities of the electrodeposited Co-P and Pt electrocatalysts on ITO were evaluated in 0.5 M H_2_SO_4_ (pH ≈ 0.45) using Ag/AgCl and Pt wire as the reference and counter electrodes, respectively. Cyclic voltammetry (CV), linear sweep voltammetry (LSV), electrochemical impedance spectroscopy (EIS), and chronopotentiometry were conducted using an AMETEK potentiostat. All potentials for the electrochemical measurements were calibrated with respect to the reversible hydrogen electrode (RHE) using the following equation: *E*_RHE_ = *E*_Ag/AgCl_ + 0.197 + 0.059 × pH. LSV curves were recorded at a scan rate of 10 mV s^−1^. The EIS measurements were conducted in the frequency range of 10^5^–1 Hz at a potential of −0.2 V vs. RHE with an AC potential amplitude of 5 mV. The CV curves were measured in a non-Faradaic region (0.2 to 0.27 vs. RHE) at a scan rate of 20–200 mV s^−1^ to obtain electrochemical double-layer capacitance. The chronopotentiometry measurements were performed at a fixed current density of 10 mA cm^−2^ for 12 h to evaluate the durability of the electrodeposited Co-P electrocatalysts.

## 3. Results and Discussion

### 3.1. Structural Evolution of Co-P Nanostructures

The Co-P nanostructures were synthesized based on the following reaction (Equation (1)) during the electrodeposition process [28,29].

Co^2+^ + H_2_PO_2_^−^ + 2H^+^ + 3e^−^ → Co-P + 2H_2_O (1)

Electrodeposition was performed on ITO using chronoamperometry for 10 min. To determine the optimal potential windows, the applied voltages were varied, and the growth of Co-P nanostructures was observed from −0.9 V. FE-SEM images in Figure 1 show the morphologies of electrodeposited Co-P under different applied potentials. At −0.9 V, cabbage-like Co-P nanosphere assemblies with an average size of 700 ± 200 nm are formed (Figure 1c,g). When the applied voltage is increased to −1.0 V, that is, the more cathodic potential is provided to the reactants, a decrease in the size and vertical growth of the Co-P nanoclusters is observed (Figure 1d,h). The color change of the Co-P film to black at −1.0 V (Figure 1b) indicates the protrusion of Co-P nanoclusters in the direction normal to the substrate [30,31,32]. During the electrochemical deposition at −1.1 and −1.2 V, the Co-P nanopillar arrays disappear; however, raspberry-like Co-P nanoclusters comprising smaller Co-P nanoparticles remain (Figure 1e,f,i,j). The variation in the darkness of the Co-P film at different applied potentials (Figure 1b) indicates that the surface coverage or the density of the Co-P nanoclusters on ITO depends on the applied potentials. Further, uniform deposition is observed up to −1.0 V.

### 3.2. Analysis of the Chemical Composition of Co-P Nanostructures

**Figure 2:**
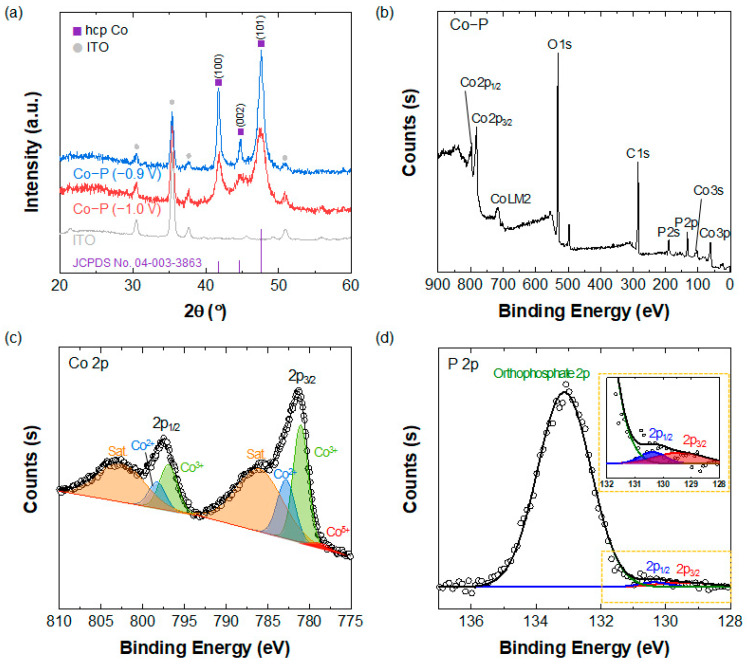
(**a**) XRD patterns of Co-P electrodeposited at applied voltages of −0.9 and −1.0 V on ITO substrate. (**b**–**d**) XPS spectra of Co-P prepared at −1.0 V. (**b**) Survey scan and high-resolution spectra of (**c**) Co 2p and (**d**) P 2p of the Co-P nanoclusters.

XRD patterns of Co-P nanostructures that were electrodeposited on bare ITO substrate at applied potentials of −0.9 and −1.0 V are obtained, as shown in Figure 2a. Three distinct diffraction peaks are observed at 41.8°, 44.8° and 47.6°, which can be assigned to the (100), (002), and (101) planes of hexagonal close-packed Co (JCPDS no. 04-003-3863). No diffraction peaks related to the polymorphs of Co-P are observed, presumably owing to the nature of the electrodeposition technique, which occasionally results in a lower portion of hybridization [28]. However, the XPS spectra of the Co-P nanostructures in Figure 2b–d confirm the existence of Co-P. The high-resolution spectrum of Co 2p in Figure 2c shows a small peak at 778.9 eV, which is attributed to the positively charged Co^δ+^ species in Co-P, in addition to the peaks from the oxidized Co state (Co^2+^ and Co^3+^) and the satellite features (Sat) [23,33,34,35,36]. The high-resolution XPS spectrum of P 2p in Figure 2d shows two peaks for P 2p_3/2_ and P 2p_1/2_ of Co-P at 129.4 and 130.4 eV, respectively, ref. [37] and also an orthophosphate peak at 133.1 eV, which indicates the formation of Co_3_(PO_4_)_2_ [38].

### 3.3. HER Performance of Co-P Electrocatalysts

**Figure 3:**
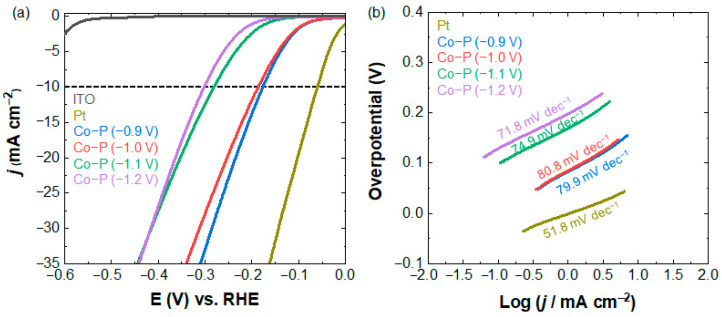
(**a**) HER polarization curves and (**b**) corresponding Tafel plots of electrodeposited Co-P and Pt electrocatalysts on ITO substrate.

As shown in Figure 3a, the HER activities of the Co-P nanostructures are evaluated by LSV in a 0.5 M H_2_SO_4_ aqueous solution. The overpotentials to reach the current density (*j*) of 10 mA cm^−2^ were increased in the following order: Co-P nanostructures fabricated under the electrodeposition at −1.2 V (302.9 mV) > −1.1 V (281.2 mV) > −1.0 V (186.9 mV) > −0.9 V (176.5 mV), confirming that the Co-P electrocatalysts prepared at −0.9 V showed the best HER performance. Metallic Pt nanoparticles evenly distributed on ITO (Figures S1 and S2) were prepared using the same electrodeposition technique and were used to estimate the HER performance of Co-P. The Pt electrocatalysts exhibited a higher HER performance than Co-P, with an overpotential of 60.2 mV at *j* = 10 mA cm^−2^. 

**Figure 4:**
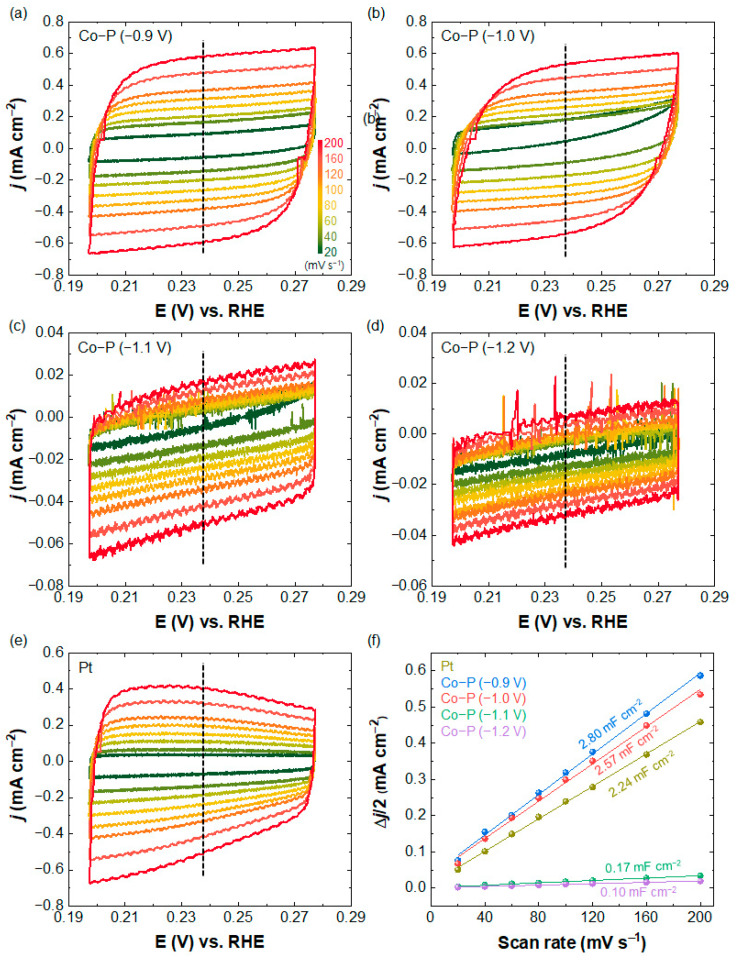
CV curves of (**a**–**d**) Co-P and (**e**) Pt electrocatalysts prepared at various applied potentials. Scan rates were controlled in the range of 20–200 mV s^−1^. (**f**) Calculated *C*_dl_ for Co-P and Pt electrocatalysts at *j*_a_ and *j*_c_ of 0.237 V vs. RHE (marked as dashed lines in the cyclic voltammograms of (**a**–**e**)).

To determine the origin, we estimated the ECSA of the Co-P nanostructures. Because ECSA is expressed using the electrochemical double-layer capacitance (*C*_dl_) (ECSA = *C*_dl_/*C*_S_; *C*_S_, which refers to the specific capacitance of an electrode with flat surface (35 μF cm^−2^) [39,40,41,42,43,44,45]), we performed CV to obtain *C*_dl_ in the non-Faradaic region (Figure 4a–e). The slopes of the linear fit of Δ*j*/2 = (*j*_a_ − *j*_c_) (*j*_a_ and *j*_c_ are the anodic and cathodic current densities at 0.237 V vs. RHE, respectively, in the cyclic voltammograms) vs. the scan rate (*V*_b_) (Figure 4f) were *C*_dl_ (*C*_dl_ = *d*(Δ*j*)/2*dV*_b_). *C*_dl_ for Co-Ps electrodeposited at −0.9, −1.0, −1.1, and −1.2 V were 2.80, 2.57, 0.17, and 0.10 mF cm^−2^, respectively, and the corresponding ECSA were 29, 24, 1.7, and 0.9 cm^2^, which is in good agreement with the LSV results. The ECSA values of Co-P grown at −0.9 and −1.0 V were higher than that of Pt electrocatalysts (23.8 cm^2^), indicating that the Co-Ps possess a substantial number of effective sites for HER. In particular, the Co-P nanostructure prepared at −0.9 V showed a higher HER performance than the hierarchical vertical assembly of Co-P nanospheres grown at −1.0 V. The degradation of electrocatalytic activity may be attributed to structural instability. The SEM images in Figure S3 show that the one-dimensional Co-P nanoclusters disassembled after the LSV experiment, whereas the shapes and the sizes of the Co-P nanostructures grown at −0.9 V were preserved. The less crystallinity of Co-P may be another implication of the HER activity (Figure 2a).

**Figure 5:**
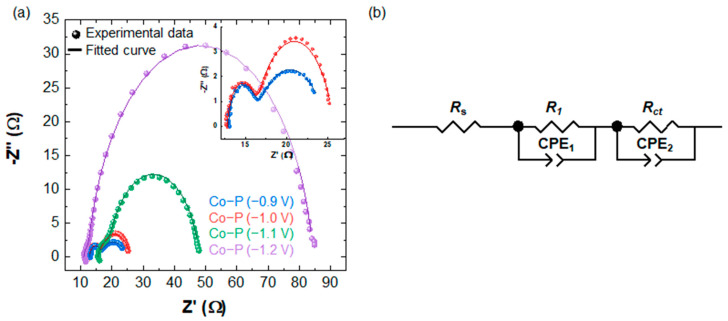
(**a**) Nyquist plots and (**b**) equivalent circuit models of the electrodeposited Co-P electrocatalysts. In (**a**), the impedance spectra were recorded at −0.2 V vs. RHE. Solid symbols and lines represent the raw data and fitted curves, respectively.

Figure 5a shows the Nyquist plots of electrodeposited Co-P electrocatalysts recorded at −0.2 V vs. RHE and fitted according to the proposed equivalent circuit model shown in Figure 5b (*R*_s_ and CPE represent the series resistance and constant phase elements, respectively. *R*_1_ refers to the series resistance at the interface between the substrate and electrocatalyst, and *R*_ct_ indicates the charge-transfer resistance at the interface between the electrocatalyst and electrolyte). Compared with the Co-P electrocatalysts prepared at −1.1 and −1.2 V exhibiting significant *R*_ct_ values of 29 and 72 Ω, the *R*_ct_ values of the Co-Ps substantially decreased to 6.9 and 9.5 Ω when the applied potentials for the electrodeposition were reduced to −0.9 and −1.0 V, respectively. The lower *R*_ct_ values of Co-P grown at −0.9 V may also cause a higher HER performance.

Tafel analysis was performed to assess the underlying mechanisms of the HER by Co-P electrocatalysts, and the results are shown in Figure 3b. In acidic media, HER can occur via the following reactions [42,46,47]:

Discharge step (Volmer reaction): H_3_O^+^ + e^−^ + M → M–H + H_2_O

Desorption step (Heyrovsky reaction): M–H + H_3_O^+^ + e^−^ → H_2_ + H_2_O + M

Recombination step (Tafel reaction): 2M–H → 2M + H_2_

where M and M–H refer to the catalytic active sites and catalytic surfaces covered with adsorbed hydrogen atoms, respectively. In acidic media, the adsorption of protons from the hydronium ions (H_3_O^+^) to M to generate metal hydride occurs first (Volmer reaction), followed by the HER through either the electrochemical Heyrovsky or chemical Tafel reactions. Tafel slopes of 70–80 mV dec^−1^ for electrodeposited Co-P and ~52 mV dec^−1^ for Pt (Figure 3b) confirm that the Volmer–Heyrovsky process was dominant for water electrocatalysis in our study. The HER Tafel slopes of the electrodeposited Co-P are comparable to previously reported values [28,48,49].

**Figure 6:**
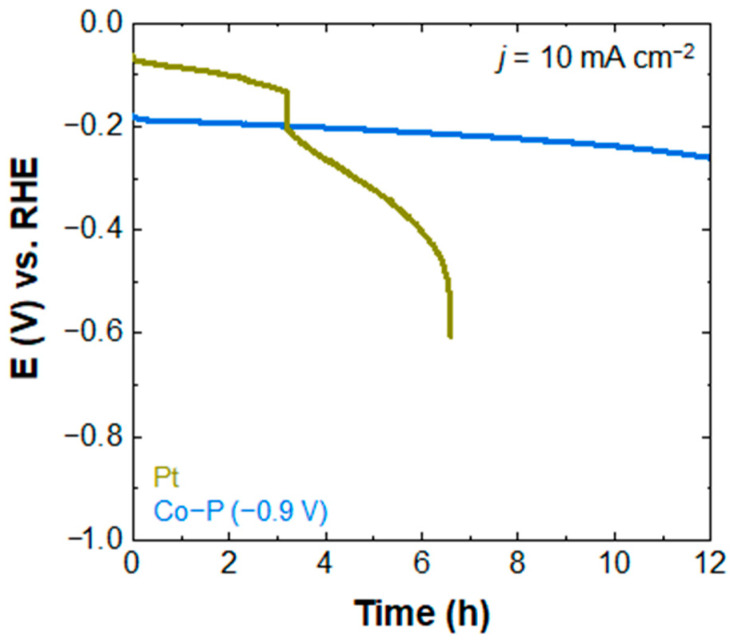
Chronopotentiometry curves of Co-P electrodeposited at −0.9 V, and Pt on ITO substrate at a current density of 10 mA cm^−2^.

Further, chronopotentiometry measurements were performed at a fixed current density of 10 mA cm^−2^ to test the durability of the electrodeposited Co-P at −0.9 V and Pt electrocatalysts. In Figure 6, a slight potential drop is observed for the Co-P electrocatalysts during operation for 12 h, while Pt shows a complete loss of its electrocatalytic activity in approximately 6 h. This result indicates that Co-P electrocatalysts may outperform Pt in terms of their long-term stability.

## 4. Conclusions

Hierarchical Co-P nanostructures were fabricated via one-step electrodeposition techniques for the electrocatalytic HER. The applied potentials during electrodeposition determined the morphologies and coverages of Co-P. The HER performance of the non-noble metal electrocatalysts was evaluated in acidic media. Co-P grown at −0.9 V exhibited the lowest overpotential at 10 mA cm^−2^ owing to its superior crystallinity, electrochemical surface area, and charge transfer characteristics. The electrodeposited Co-P also demonstrated long-term stability over the Pt electrocatalysts. To further ameliorate the electrocatalytic activity of the Co-P nanostructures, we plan to introduce additional layers, such as porous carbonaceous sheaths, to improve the conductivity and the durability of the electrocatalysts.

## Data Availability

Not applicable.

## Supplementary Materials

The following supporting information can be downloaded at: https://www.mdpi.com/article/10.3390/ma16020593/s1, Figure S1: SEM images of Pt on ITO which were electrodeposited at an applied voltage of −0.35 V for 10 min.; Figure S2: (a) XPS survey and (b) Pt 4f spectra of Pt.; Figure S3: SEM images of Co-P on ITO (a,b) before and (c,d) after HER experiments in aqueous 0.5 M H_2_SO_4_. The applied voltages during the electrodeposition growth of the Co-P nanostructures were (a,c) −0.9 V and (b,d) −1.0 V. Reference [50] is cited in the supplementary materials.
