# Supplementary material for: Electrodeposition of Stable Noble-Metal-Free Co-P Electrocatalysts for Hydrogen Evolution Reaction
Source: Materials (Basel). 2023 Jan 7;16(2):593. doi: 10.3390/ma16020593 (PMC9867289; doi:10.3390/ma16020593)
Supplement: Supplementary file 1 [file materials-16-00593-s001.zip › materials-2053029-supplementary.pdf]

materials

Communication

Electrodeposition of Stable Noble-Metal-Free Co-P Electrocatalysts for Hydrogen Evolution Reaction

Jeongwon Kim [1], Yu Jin Jang [2,*] and Yoon Hee Jang [1,*]

[1] Advanced Photovoltaics Research Center, Korea Institute of Science and Technology (KIST), Seoul 02792, Republic of Korea
[2] Convergence Research Center for Energy and Environmental Sciences, Sungkyunkwan University (SKKU), Suwon 16419, Republic of Korea
* Correspondence: jllv@skku.edu (Y.J.J.); yhjang@kist.re.kr (Y.H.J.)

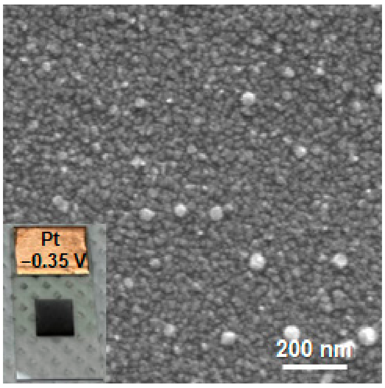

Figure S1. SEM images of Pt on ITO, which were electrodeposited at an applied voltage of −0.35 V for 10 min.

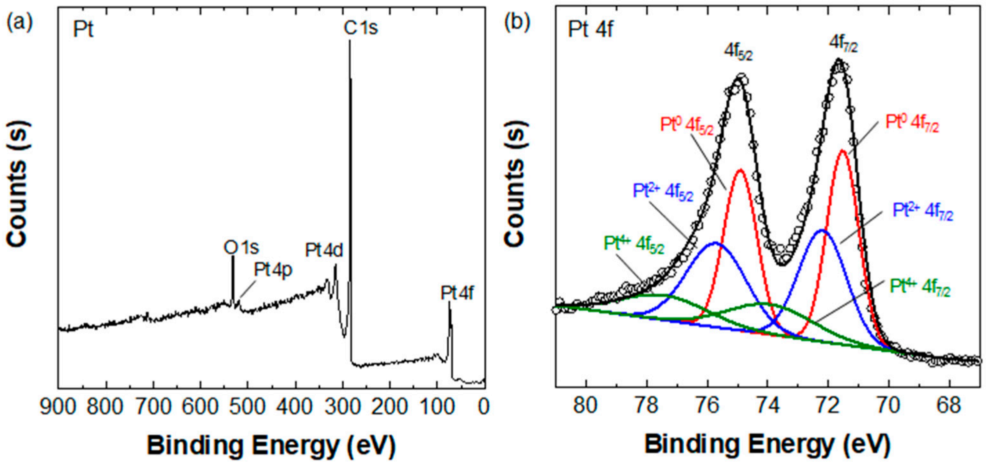

Figure S2. (a) XPS survey and (b) Pt 4f spectra of the electrodeposited Pt nanostructures. High-resolution Pt 4f spectra in Figure S4b show the peaks centered at 71.5 and 74.9 eV, which correspond to Pt^0 $4f_{7/2}$ and Pt^0 $4f_{5/2}$ of the metallic Pt. Other peaks associated with PtO (Pt^{2+} $4f_{7/2}$ and Pt^{2+} $4f_{7/2}$,) and PtO_2 (Pt^{4+} $4f_{7/2}$ and Pt^{4+} $4f_{5/2}$) are also observed [50].

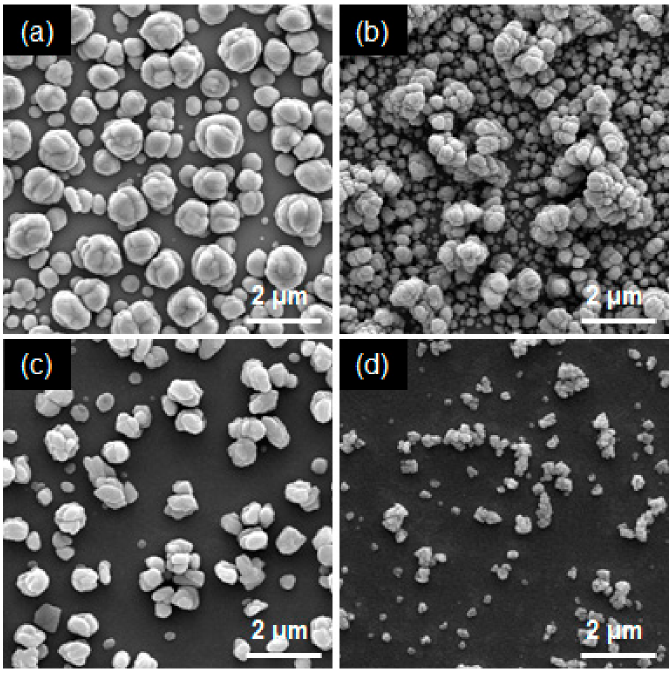

Figure S3. SEM images of Co-P on ITO (a and b) before and (c and d) after HER experiments in aqueous 0.5 M H_2SO_4. The applied voltages during the electrodeposition growth of Co-P nanostructures were (a and c) −0.9 and (b and d) −1.0 V.
